# Lung ultrasound in internal medicine: training and clinical practice

**DOI:** 10.1186/s13089-016-0048-6

**Published:** 2016-08-08

**Authors:** Chiara Mozzini, Anna Maria Fratta Pasini, Ulisse Garbin, Luciano Cominacini

**Affiliations:** Department of Medicine, Section of Internal Medicine, University of Verona, Piazzale L.A. Scuro, 10, 37134 Verona, Italy

**Keywords:** Lung ultrasound (LUS), Internal Medicine, Training, Clinical practice

## Abstract

**Background:**

Lung ultrasound (LUS) represents an emerging technique for bedside chest imaging in different clinical settings. A standardized approach allows the diagnosis, the quantification, and the follow-up of different conditions for which acute respiratory failure is the main clinical presentation. The aim of this study was to test what skill targets could be achieved in LUS, with a short-training course offered to 19 Medical Doctors attending the certification board school in Internal Medicine at the University of Verona, Italy.

**Methods:**

The training course (theoretical and practical) consisted of 9 h subdivided in 4 days. Each trainee examined three healthy volunteers during the first day that was also the day of the theoretical lessons. Moreover, they examined nine patients per day (a total of 27 patients). Trainees were tested in the recognition of the basic signs in LUS, the managing of the Bedside Lung Ultrasound Evaluation (the BLUE protocol), and the recognition of the broad clinical scenarios recognized by the LUS. Kappa statistic was used to calculate the inter-observer agreement (trainees/tutor).

**Results:**

Twenty-seven patients were examined by the 19 trainees (ten trainees had previous limited experience in general ultrasound). The agreement among the trainees and the tutor in the recognition of the LUS basic signs and in the recognition of the BLUE protocol profiles ranged from “fair” to “excellent”. In particular, the agreement among the trainees and the tutor in the final LUS diagnosis was “excellent” for the recognition of the interstitial syndrome and the pleural effusion, “substantial” for the recognition of the normal lung, and “moderate” for the recognition of consolidation and pneumothorax. LUS outcome gave useful information and drove change in therapy in 16 patients. It affected immediate management in nine patients. The concordance between the previous X chest ray and LUS was observed in 21 patients.

**Conclusions:**

A short training in LUS provided good proficiency in the recognition only of the main signs of the BLUE protocol, but allowed a correct LUS diagnosis in the Internal Medicine most frequent clinical settings of acute respiratory failure. This study supports incorporating LUS into Internal Medicine fellowship training programs.

## Background

The evaluation of the lungs has been considered an “off-limits” area for ultrasound for many years, considering the air as the main obstacle. Nevertheless, in the last years, lung ultrasound (LUS) has emerged in different clinical settings, such as in emergency and critical care medicine [1–3], but also in cardiology [4] and in Internal Medicine [5]. LUS represents an adjunct to the physical examination to recognize specific ultrasonic signs that represent a narrow list of potential diagnoses in specific clinical settings [6–8]. A standardized approach allows the diagnosis, the quantification, and the follow-up of different conditions for which acute respiratory failure is the main clinical presentation. In particular, the main fields of LUS applications are: the pneumothorax (PNX), the interstitial syndrome, the lung consolidation, and the pleural effusion [6–8]. Recently, the World interactive network focused on critical ultrasound (WINFOCUS) has proposed a document [9] reporting evidence-based recommendations on the clinical use of LUS. LUS uses the basic technology (only 2D and M-mode are required and every probe can be appropriate), it is free of ionizing radiations, and it is not invasive, repeatable and effective. It does not require transfer of patients [10]. Nevertheless, it is largely operator dependent, and a scientific assessment of the learning curve remains to be assessed [11]. The main limitation of LUS is that it does not rule out any pulmonary abnormality that does not reach the pleura [10].

The primary aim of this study was to test what skill targets could be achieved in LUS. A relatively short-training course was offered to already certified Medical Doctors attending the certification board school in Internal Medicine. Part of this aim was established more precise rules in training courses based on the previous expert consensus recommendations [9]. The desired level of competence consisted in acquiring technical skills and interpretative skills. The essence of LUS is mainly a dichotomous interpretation of the findings. The diagnostic approach is qualitative or semi-quantitative. The objectives were: the recognition of the basic signs in LUS, the managing of the bedside lung ultrasound evaluation (the BLUE protocol) [12, 13], and the recognition of the broad clinical scenarios recognized by LUS [8]. The second aim of this study was to depict the clinical utility of LUS in an Internal Medicine department. The goal was to show the usefulness of LUS in the management of the most common causes of acute respiratory failure in this department.

## Methods

### Participants

The study setting was the Internal Medicine department of the University Hospital of Verona, Italy, already certified as a first level ultrasound centre by the Società Italiana di Medicina Interna (SIMI). Nineteen Medical Doctors attending the certification board school in Internal Medicine at the University of Verona accepted to follow a short-training course in LUS. The training course consisted of a total of 9 h of theoretical and practical lessons over a period of 4 days. The tutor was a general ultrasound certified Internal Medicine specialist with the formal competency from the Società Italiana di Ultrasonologia in Medicina e Biologia (SIUMB). A subgroup of the trainees had previous limited theoretical and practical experience in general ultrasound (basic principles of ultrasound and basic knowledge of the devices) and in focused cardiac ultrasound (no competence certification, nor daily practice of ultrasound).

### Ethics statement

The study was conducted in accordance with the ethical standards laid down in the Helsinki Declaration of 1975 and its late amendments. The participants provided written consent prior to starting the study (for collecting and publishing data). The procedure did not require a particular approval by the local Ethical Committee.

### Description of the training and evaluation program

First, the tutor did theoretical lessons of 2 h and 20 min which included these precise lectures: introduction, contents, advantages, and disadvantages of LUS (5 min); brief theoretical summary of the basic principles of ultrasound and of the characteristics of the device used (EnVisor C HD, Philips, equipped with linear, convex, and sector transducers) (5 min); the international evidence-based recommendations for point of care lung ultrasound [9] (15 min); and the flow chart of different diagnoses according to the BLUE protocol [12, 13] (15 min). Particular attention was paid to the patient’s position and to the correct scanning technique, as previously recommended [6, 7, 9]: the eight-zone examination modality (15 min); the twenty-eight scanning site scheme (10 min); and the possibility of a more rapid two-region scan as an option for the critically ill patients (5 min). Photos and video clips were used to demonstrate the LUS signs and patterns. The signs of the BLUE protocol [12, 13] were explained (30 min): bat sign; lung sliding; seashore sign (in M-mode); A lines (normal lung tissue yields, lung sliding, and horizontal repetition artifacts arising from the pleural line); quad sign; sinusoid sign; fractal sign; tissue-like sign; stratosphere sign; lung point (M-mode); lung pulse; and dynamic air bronchogram, and B lines (artifacts that correlate with interstitial edema, and, if three or more between two ribs, are called lung rockets). Then, the profiles of the BLUE protocol derived from the association of the signs were explained, and the BLUE protocol “decision tree” [12, 13] was analysed (10 min): A profile; A’ profile; B profile; B’ profile; C profile; and A/B profile. Then, according to the international recommendations [9], the main clinical scenarios were discussed (30 min): normal lungs; PNX; interstitial syndrome; and lung consolidation. Then, in the first session for each group, the training course was performed with healthy volunteers, tested as “without any lung abnormalities” by the trainer (three examinations for each trainees). In the other sessions, patients enrolled from the same Internal Medicine department were studied. Didactic material for the theoretical lectures was provided (bibliographic indexes, online material) to invite the trainees to study also on their own. During the practical sessions, each theoretical point was discussed in depth, with the aim of refreshing and fixing the main concepts. The desired level of competence consisted in acquiring technical skills (the recognition of the LUS signs and the different patterns) and in making the correct focused diagnosis. The time allotted for each examination and diagnosis making was a maximum of 3 min, according to the BLUE protocol. The trainees were blinded to the LUS examination report previously made by the tutor. The sector probe was the first choice of use, but the convex and the linear ones were also available. No specific attendance certification was issued.

The inter-observer agreement was assessed with kappa statistics based on Cohen and Fleiss’ works [14, 15]. The strength of agreement of kappa coefficients was guided by the boundaries suggested by Landis and Koch [16].

## Results

The study was conducted in Spring and Autumn 2014. Nineteen certified Medical Doctors attending the certification board school in Internal Medicine accepted to participate on the training program. The trainees were subdivided into four groups of five or four students for each one. The training course (theoretical and practical) consisted of 9 h subdivided in 4 days (3 h on the first day and 2 h on the following days). Each trainee examined three healthy volunteers during the first day that was also the day of the theoretical lessons. Moreover, they examined nine patients per day (a total of twenty-seven patients were examined) blinded to each other as regarding the final diagnosis. The patients were not selected on the basis of the difficulty in acoustic windows images acquisition or on the basis of the diagnosis difficulty. They were selected on the basis of their actual needs.

The average time to acquire and interpret LUS was tested only when patients were examined, while free time was allowed for healthy volunteer’s examination. Baseline demographics of the patients and the indications to perform LUS examination are reported in Table 1.

**Table 1:** Baseline characteristics of the examined patients and indications requested by physicians to perform the examination

Characteristics/indications	*N* = 27 patients
Median age (years)	71 ± 5
Male/female	15/12
Obese: BMI (kg/m^2^) ≥30	5
Day of the examination from the admission day	2 ± 1
Acute respiratory failure as indication	12
Short time follow-up in already diagnosed pathology as indication	10
Guide for thoracentesis/exclusion of PNX after thoracentesis	3
Exclusion of PNX after central venous catheter placement	2
X chest ray at the admission (in the Emergency Medicine department)	24

*BMI* body mass index

The agreement (*k*) and the strength of agreement between the trainees and the tutor in the recognition of the LUS basic signs, in the recognition of the profiles of the BLUE protocol, and in the final LUS diagnosis are displayed in Tables 2, 3, and 4.

**Table 2:** Agreement between the trainees and the tutor in the recognition of the LUS basic signs

LUS basic signs (recognition of)	Students without any previous experience in ultrasound (*n* = 9)			Students with previous limited experience in Cardiac ultrasound (*n* = 10)		
	*k*	95 % CI	Agreement strength	*k*	95 % CI	Agreement strength
The bat sign	0.30	0.28–0.33	Fair	0.28	0.26–0.31	Fair
The lung sliding	0.71	0.65–0.79	Substantial	0.77	0.73–0.80	Substantial
The seashore sign (M-mode)	0.45	0.41–0.49	Moderate	0.70^a^	0.68–0.72	Substantial
The A lines	0.90	0.81–1.00	Excellent	0.91	0.81–1.00	Excellent
The quad sign	0.33	0.29–0.40	Fair	0.28	0.25–030	Fair
The sinusoid sign	0.44	0.41–0.46	Moderate	0.44	0.41–0.46	Moderate
The fractal sign	0.30	0.27–0.32	Fair	0.35	0.28–0.40	Fair
The tissue-like sign	0.80	0.78–0.82	Substantial	0.80	0.79–0.81	Substantial
The B lines recognition	0.90	0.88–0.92	Excellent	0.90	0.86–0.94	Excellent
The B lines quantification	0.70	0.67–0.73	Substantial	0.72	0.70–0.74	Substantial
The lung rockets	0.50	0.48–0.52	Moderate	0.52	0.51–0.53	Moderate
The stratosphere sign (M-mode)	0.80	0.78–0.82	Substantial	0.84^a^	0.82–0.84	Excellent
The lung point	0.80	0.78–0.82	Substantial	0.79	0.78–0.80	Substantial
The lung pulse	0.80	0.80–0.80	Substantial	0.78	0.77–0.79	Substantial
The dynamic air bronchogram	0.78	0.77–0.79	Substantial	0.76	0.73–0.79	Substantial

*CI* confidence intervals

^a^Significantly different between the two groups

**Table 3:** Agreement between the trainees and the tutor in recognition of the BLUE protocol profiles

The profiles of the BLUE protocol (recognition of)	Students without any previous experience in ultrasound (*n* = 9)			Students with previous limited experience in cardiac ultrasound (*n* = 10)		
	*k*	95 % CI	Agreement strength	*k*	95 % CI	Agreement strength
The A profile	0.77	0.73–0.80	Substantial	0.71	0.61–0.80	Substantial
The A’ profile	0.76	0.75–0.77	Substantial	0.76	0.74–0.78	Substantial
The B profile	0.77	0.76–0.78	Substantial	0.75	0.74–0.76	Substantial
The B’ profile	0.72	0.70–0.72	Substantial	0.72	0.66–0.78	Substantial
The C profile	0.32	0.30–035	Fair	0.35	0.29–0.41	Fair
The A/B profile	0.50	0.48–0.52	Moderate	0.51	0.49–0.53	Moderate

*CI* confidence intervals

**Table 4:** Agreement between the trainees and the tutor in the final LUS diagnosis

Final LUS diagnosis	Students without any previous experience in ultrasound (*n* = 9)			Students with previous limited experience in cardiac ultrasound (*n* = 10)		
	*k*	95 % CI	Agreement strength	*k*	95 % CI	Agreement strength
The normal lung	0.65	0.61–0.69	Substantial	0.66	0.61–0.72	Substantial
The pneumothorax	0.46	0.45–0.47	Moderate	0.48	0.46–0.50	Moderate
The interstitial syndrome	0.82	0.81–0.83	Excellent	0.84	0.82–0.86	Excellent
The consolidation	0.45	0.44–0.46	Moderate	0.48	0.47–0.49	Moderate
The pleural effusion with the correct recognition of its nature (transudates/exudates)	0.82	0.81–0.83	Excellent	0.83	0.82–0.84	Excellent

*CI* confidence intervals

The agreement among the trainees and the tutor in the recognition of the LUS basic signs and in the recognition of the BLUE protocol profiles ranged from “fair” to “excellent”. In particular, the agreement among the trainees and the tutor in the final LUS diagnosis was “excellent” for the recognition of the interstitial syndrome and the pleural effusion, “substantial” for the recognition of the normal lung, and “moderate” for the recognition of consolidation and pneumothorax.

Table 5 shows the LUS outcome for the examined patients (the results were reported on the basis of the findings of the formal examination performed by the tutor). LUS outcome gave useful information and drove change in therapy in 16 patients. It affected immediate management in nine patients. The concordance between previous X chest ray and LUS was observed in 21 patients.

**Table 5:** LUS outcome for the examined patients

LUS outcome	*N* = 27 patients
Affected immediate management	9
Gave useful information and drove change in therapy	16
No useful information nor change in therapy	2
Concordance between previous X chest ray and LUS	21
X chest ray examination or thoracic computed tomography need to confirm the management	4

*LUS* lung ultrasound

Changing in therapy or management included: diuretic therapy dosage improvement or lowering, antimicrobial therapy insertion, rapid fluid administration, thoracic surgeon consultation, thoracentesis execution, thoracic computed tomography examination, patient’s discharge from the Internal Medicine department, and their admission to the Intensive Care Unit.

Table 6 depicts the final discharge diagnosis (with respect to lung findings) cited in the discharge report from the department. For some patients, the coexistence of two or more pathologies was reported. Heart failure, pneumonia, and pleural effusion were the most common final discharging diagnosis.

**Table 6:** Summary of the final discharging diagnosis (for some patients two or more diagnosis were reported)

Discharging diagnosis cited in the final report	*N* = 27 patients
Heart failure	22
Pneumonia	12
Pleural effusion	10
PNX	2
Pulmonary fibrosis	1
ARDS	1

*ARDS* acute respiratory distress syndrome, *PNX* pneumothorax

## Discussion

This study has been designed to assess the feasibility and the clinical utility of a short-training course in LUS for Internal Medicine certification board school attending Medical Doctors. The primary aim was to collect experience that could promote the assessment of systematic standards in training courses. Nowadays, the absence of appropriate and validated teaching models and programs is a great obstacle in this area. The training course has been precisely structured, starting with lectures (photos and video clips) and then hands-on training. The model to start with normal volunteers seems to be a convenient and effective method to teach the key elements of images acquisition, ability in probe manipulation, and spatial orientation. The beginning was the exploration of the normal lung. The sonographic technique was conducted based only on the current international evidence-based recommendations [9], focusing in particular on the BLUE protocol [12, 13]. This choice was to standardize knowledge and skills. The results of the present study suggest an acceptable ability level in performing LUS and in allocating the patients in one of its principal clinical scenarios. However, some tasks were more difficult to acquire and interpret. In general, there was not a significant difference in performing LUS on the basis of previous knowledge and practice in cardiac ultrasound, except for obese patients (data not analysed). The same results for the detection of those signs for which M-mode evaluation is required. The lack of substantial difference could be due to the fact that the trainees with previous knowledge in focused cardiac ultrasound had limited experience and practice. Therefore, these data confirm that LUS is relatively easy to perform even in non-ultrasound-experienced physicians [11, 17]. Regarding the average time to acquire and interpret the LUS, the results of this training course substantially agree with the time allotted by the BLUE protocol (maximum 3 min) [12, 13]. The adherence to the BLUE protocol was mandatory for the trainer, with the aim of standardizing the procedure. More time was required (and consented) by the trainers during the management of the healthy volunteers. Regarding the training period, previous experiences [11] have shown that a 1 day course could be sufficient to acquire theoretical and practical skills in LUS. More work has to be done to standardize these training courses. The precise time to devote and the examination number to be done to have the ability to perform a useful LUS has to be identified.

Although the study was principally assessed to evaluate a model of LUS training, some remarks could be made about the indications to perform LUS. They reflect the most common indications in an Internal Medicine department. In particular, heart failure is one of the most common admission diagnoses. In this context, the main sign that has to be considered is the presence of B lines. They surely represent an easy to acquire and highly reproducible sign, but their limitation is the low specificity [18]. B lines due to cardiogenic pulmonary edema are usually diffusing or recovering symmetrically. In fact, their regular distribution allows to differentiate cardiogenic pulmonary edema from ARDS and from pulmonary fibrosis [2]. This last is also characterized by irregularities of the pleural line and sub-pleural small consolidations. The B lines have to be used both as diagnostic modality and in monitoring heart failure therapy [18]. The clearance of the B lines represents a direct sign of effective treatment in heart failure and, as we have demonstrated, a careful assessment and quantification of them is useful also in the follow-up. It has been shown [4, 18] that ultrasound accuracy has even higher sensitivity than chest X ray in the detection of the early signs of interstitial thickening due to the pulmonary congestion. Sonographic B-lines’ assessment is surely of particular interest in LUS. A recent study [19] has evaluated the agreement among trained and novice Medical Doctors in an Emergency department. Authors tried to determine which thoracic zones could represent the highest level of inter-observer reliability for B-lines’ assessment. They found that agreement was best in the anterior/superior thoracic zones, and it was highest at extreme high or low numbers of B lines. This study has to be mentioned, because training assessment in B-lines’ recognition is a promising area of investigation, relatively simple but mandatory. Moreover, the precise thoracic zone definition is a more detailed information. It is not present in our study. This fact could be a limitation of our current training program.

In addition, pneumonia represents a very common diagnosis in the Internal Medicine department, associated with increasing morbidity and mortality, hospitalization rate, and health costs [20]. Pneumonia LUS pattern is constituted by consolidations and dynamic air bronchograms. These signs could be frequently associated with pleural effusion or interstitial syndrome images [21]. It is well established that computed tomography represents the gold standard and that chest X ray remains the daily reference for lung imaging in the diagnosis of pneumonia [21]. However, recent studies have confirmed excellent sensitivity and specificity of LUS, with percentages comparable with chest X ray [18, 21, 22] (the main limitation is that LUS may only detect lesions reaching the pleura). Nevertheless, it is to mention that these comparable results could be obtained only with experienced LUS operators. Therefore, in this context, the importance of precise training courses and a common level of proficiency are mandatory. As described in “Results”, LUS gave useful information and drove change in therapy in the majority of the analysed cases. It affected immediate management in a consistent number of cases. This point underlines its importance. The LUS operator was not blinded to the patients’ clinical situation (while the Radiologist usually does not see the patients but only an information summary about them). This modality underlines the usefulness of this imaging examination. It could be performed at the bedside by the physician who is looking after the patient and who is better able to place any LUS finding in a more appropriate context with other clinical problems.

Nevertheless, this study has several limitations. The results do not address what level of experience is needed to maintain the skills. They inform only about the initial training assessment required. Second, the post-course performance of the trainees was tested just shortly after the completion of the program. This is an important point to be considered in each area of ultrasound. Trainees’ skills decrease as a function of time, particularly when ultrasound techniques are not conducted regularly and further supervised teaching is not available. Moreover, this is an Internal Medicine department experience. No patients directly derived from the emergency setting were tested. According to the admission characteristics in the Internal Medicine department, the evaluation of a sufficient number of PNX, ARDS, and artificial ventilation patients was not possible. In fact, the study reflects the real need of this department, focusing on interstitial syndrome, pleural effusion, and consolidation (both as acute and as follow-up indication for LUS). In particular, the ability to rule out the PNX was critical after procedures, such as the insertion of central venous catheter but mostly after the thoracentesis. This procedure is not so unusual for the Internal Medicine department. LUS should be mandatory both as procedure guide and as procedure monitoring to reduce the iatrogenic complications [23, 24].

## Conclusions

This study supports incorporating LUS into Internal Medicine fellowship training programs. It should facilitate the design of future training courses. The maximum effectiveness of the method is obtained through a clinically driven, focused and methodologically learned assessment. As mentioned by Lichtenstein, who significantly improved the expansion of LUS explaining his “ten good reasons to practice ultrasound in critical care” [25], it is hopeful that these good reasons could reach also the Internal Medicine care. This contributes to a widespread and routine use of LUS in this department with rigorous and scientific training courses protocols.

## Abbreviations

ARDS: acute respiratory distress syndrome
BLUE: bedside lung ultrasound evaluation
BMI: body mass index
LUS: lung ultrasound
PNX: pneumothorax
SIMI: Società Italiana di Medicina Interna
SIUMB: Società Italiana di Ultrasonologia in Medicina e Biologia
WINFOCUS: World interactive network focused on critical ultrasound
